# A Deep Neural Network-Based Pain Classifier Using a Photoplethysmography Signal

**DOI:** 10.3390/s19020384

**Published:** 2019-01-18

**Authors:** Hyunjun Lim, Byeongnam Kim, Gyu-Jeong Noh, Sun K. Yoo

**Affiliations:** 1 Department of Medical Engineering, Yonsei University College of Medicine, Seoul 03722, Korea; hhyunjjun@naver.com (H.L.); bnkim007@gmail.com (B.K.); 2 Department of Anaesthesiology and Pain Medicine, Asan Medical Center, University of Ulsan College of Medicine, Seoul 05505, Korea; nohgj@amc.seoul.kr; 3 Department of Clinical Pharmacology and Therapeutics, Asan Medical Center, University of Ulsan College of Medicine, Seoul 05505, Korea

**Keywords:** pain, photoplethysmography, deep belief network, bagging

## Abstract

Side effects occur when excessive or low doses of analgesics are administered compared to the required amount to mediate the pain induced during surgery. It is important to accurately assess the pain level of the patient during surgery. We proposed a pain classifier based on a deep belief network (DBN) using photoplethysmography (PPG). Our DBN learned about a complex nonlinear relationship between extracted PPG features and pain status based on the numeric rating scale (NRS). A bagging ensemble model was used to improve classification performance. The DBN classifier showed better classification results than multilayer perceptron neural network (MLPNN) and support vector machine (SVM) models. In addition, the classification performance was improved when the selective bagging model was applied compared with the use of each single model classifier. The pain classifier based on DBN using a selective bagging model can be helpful in developing a pain classification system.

## 1. Introduction

The pain that people experience in their lives is very diverse in cause and extent. Even when exposed to the same stimulus, some people carry out their daily activities with an acceptable level of pain, while others experience extreme pain. This phenomenon results because sensitivities to stimuli differ depending on an individual’s constitution and tendencies. Most pain occurs as a major symptom of a medical condition, but pain can also occur despite lack of any stimulation or pathological cause [1]. If such pain persists, a complex stress response is generated in the body. Persistence of this stress response can seriously affect the manner in which people live and perform basic functions [2].

The stress response experienced by individuals during surgery is an unconscious response to tissue injury and refers to autonomic, hormonal, and metabolic changes that follow injury [3]. This sustained stress during surgery leads to high mortality and delayed postoperative recovery, so analgesics are administered to control stress [4]. However, when an excessive amount of an analgesic is administered compared to the required amount to mediate the pain induced during surgery, the vital signs of the patient are excessively lowered during the surgical procedure. This phenomenon can lead to obstacles to circulation that make it difficult to maintain proper functioning of the body and may result in delayed postoperative recovery. Conversely, if analgesics are not adequately administered, stress caused by persistent pain during surgery may lead to a negative effect on the surgical outcome and postoperative aftereffects, which can result in a prolonged hospital stay and increased treatment costs. Therefore, it is important to accurately assess the pain level of anesthetized patients during surgery to allow administration of analgesics at appropriate times and levels to individual patients, maintain stable vital signs during surgery, and avoid side effects from overdosage or underdosage [5].

In hospitals, several methods are used to measure and assess patients’ pain levels. For conscious patients, the numeric rating scale (NRS) is used to measure numerical pain from no pain (0 points) to extreme pain (10 points). Pain measured with the NRS can be classified as mild (1–3 points), moderate (4–6 points), and severe (7–10 points) [6]. However, the NRS cannot be used in patients who have been anesthetized for surgery. Clinical signs of inadequate anesthesia, such as facial expressions, movements, blood pressure, and flushing, are used to assess pain levels in anesthetized patients [7]. However, as this assessment is subjective, the results are dependent on the physician’s experience, and the results are likely to vary from person to person.

This study was conducted as a preliminary study to develop a pain assessment system for patients with surgery. In this study, we present our analysis based on the time domain and frequency domain features extracted from the collected photoplethysmography (PPG) signal, which is easy to measure by minimizing the burden on the conscious patient from the status without pain in the preoperative period and the status with pain in the immediate postoperative period. In addition, we propose a technique using a deep belief network (DBN) to model the complex nonlinear relationship between the features extracted from the PPG and NRS-based pain status. DBN is a probabilistic generative model that reduces the problem of local minima through a greedy layer-wise unsupervised pre-training stage using a stack of restricted Boltzmann machines (RBMs) that initializes the weights of DBN from high dimensional input data and solves classification problems through supervised fine-tuning that adjusts weights in the direction of minimizing classification errors.

Therefore, the proposed DBN, more than the traditional models such as multilayer perceptron neural network (MLPNN) and support vector machine (SVM), is expected to show excellence as a pain classifier. In addition, the performance of the classifier is improved by using a bagging ensemble technique that combines multiple classifiers.

## 2. Materials and Methods

An experiment was conducted to acquire PPG signals in the absence of pain status at pre-operation and in the presence of pain status at post-operation. The subjects were 100 adult patients (53.8 ± 12.4 years old) scheduled for regular surgery for conditions such as gastric cancer, breast cancer, and colon cancer with no accompanying disorders that can cause spontaneous pain or affect the autonomic nervous system. This study was approved by the Asan Medical Centre Institutional Review Board (approval number: 2016-0477) and registered on an international clinical trials registry platform (http://cris.nih.go.kr, KCT0002080), and written informed consent was obtained from all patients. Subjects voluntarily agreed prior to the experiment to participate in this clinical study, and the experiment was conducted with subjects lying on a bed. First, the PPG signal was measured for 14 min in the recovery room before entering the operating room. Subsequently, general anesthesia was performed according to usual medical procedures and surgery was conducted in the operating room. At the end of the operation, the patient was awakened and moved to the recovery room. Thereafter, the PPG signal was again measured for 14 min. Every measurement was conducted at room temperature.

The PPG signals were acquired using S/5 Anesthesia Monitor (Datex-Ohmeda, Inc., Helsinki, Finland) and collected at a sampling frequency of 300 Hz. The PPG signal was measured by attaching the sensor to the left index finger. The PPG signal is versatile because it can extract various parameters such as heart rate, peripheral oxygen saturation (SpO2), respiration rate of electrocardiogram (ECG), and respiration signal indicating the autonomic nervous system response [8]. We used a PPG signal that is easy to measure and minimizes the burden on the patient by attaching it to one finger because the adhesive pads of the ECG and belt devices of respiration are known to be uncomfortable owing to movement restriction, and their measurement is limited in actual surgical situations.

During the experiment, the subjects were interviewed using the NRS (0-no pain to 10-most pain) to determine the current status of pain. The subjects’ responses were used to confirm and assess the level of pain in each situation. In the preoperative period, all 100 subjects reported no pain (0 points), and it was confirmed that almost all subjects felt no pain. In the immediate postoperative period, there was variation in the pain status of the subjects (Table 1). The 11-level of pain classification using NRS is statistically insignificant because of the small number of data points for each level. If the NRS score is more than 4 points, indicating moderate or severe pain, active treatment is considered necessary for the patient [9]. Therefore, we designed a model to classify no pain status and pain status requiring active treatment for appropriate administration of analgesics to anesthetized patients. We selected 78 subjects who reported moderate and severe pain in immediate postoperative period for analysis. We classified the data acquired in the preoperative period as no pain status (N) and that in the immediate postoperative period as pain status requiring active treatment (P). Additionally, we tried to classify the 4-class pain status—no pain status in the preoperative period and mild pain status, moderate pain status, severe pain status in immediate postoperative period. The data were analyzed using MATLAB (R2016a release, MathWorks, Inc., Natick, MA, USA).

**Figure 1:**
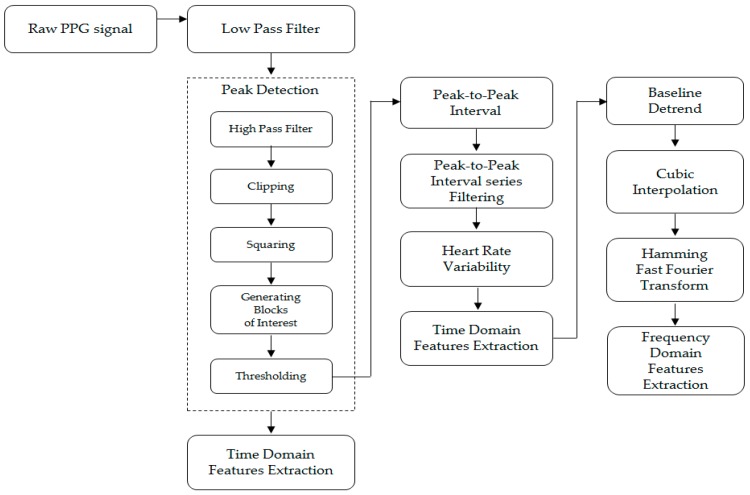
PPG signal processing and feature extraction process.

Amplitude changes of the waveform in the PPG signal detected by the sensor represent blood volume changes synchronized to each heartbeat [10]. As the response of the autonomic nervous system to external stimuli reflects cardiovascular disease, emotional status, etc., clinically significant parameters such as heart rate and heart rate variability (HRV) have been extracted from the PPG signal [8]. In this study, the signal processing algorithm is designed to extract the features that determine the accurate pain status using the PPG signal (Figure 1).

Since the measured PPG signal acquired from the operating room or the recovery room is considerably vulnerable to noises introduced from a variety of patient monitoring equipment, surgery assist devices, and power sources, a second-order Butterworth low pass filter with a cut-off frequency of 8 Hz is used to remove such noises. In order to calculate the peak-to-peak interval representing the heartbeat period, a technique to detect systolic peaks from the filtered PPG signal is required. Peaks that are local maxima of the PPG signal are extracted using the systolic peak detection algorithm proposed by Elgendi [11]. A second-order Butterworth high pass filter with a cut-off frequency of 0.5 Hz for removing baseline variation was applied to the noise filtered PPG signal. The high pass filtered PPG signal (H[n]) was negatively filtered by the clipped signal (C[n]) (Equation (1)). A squared signal (S[n]) was calculated to emphasize the peak component from the clipped signal (C[n]) (Equation (2)).

(1) C[n]=max(0,H[n])

(2) $$\left[n\right]={\left(C\left[n\right]\right)}^{2}$$

A Moving Average method was used to calculate the block of interest including the peak point from the squared signal (S[n]) for the vasoconstriction interval and heart rate cycle interval. The first moving average (${\mathrm{MA}}_{\mathrm{Peak}}$) emphasized the vasoconstriction interval (Equation (3)) and the second moving average (${\mathrm{MA}}_{\mathrm{Beat}}$) emphasized the heartbeat interval (Equation (4)). $W_{1}$ and $W_{2}$ mean the window size of the vasoconstriction interval and the heartbeat interval, which means 111 msec and 667 msec, respectively.

(3) $${\mathrm{MA}}_{\mathrm{Peak}}\left[n\right]=\frac{1}{W_{1}}(S\left[n-\frac{W_{1}-1}{2}]+\cdots +S\left[n\right]+\cdots +S[n+\frac{W_{1}-1}{2}\right])$$

(4) $${\mathrm{MA}}_{\mathrm{Beat}}\left[n\right]=\frac{1}{W_{2}}(S\left[n-\frac{W_{2}-1}{2}]+\cdots +S\left[n\right]+\cdots +S[n+\frac{W_{2}-1}{2}\right])$$

The adaptive threshold is obtained to detect the peak through two moving averages (Equations (5) and (6)).

(5) $${\mathrm{Threshold}}_{1}={\mathrm{MA}}_{\mathrm{Beat}}\left[n\right]+0.02\bar{S\left[n\right]}$$

(6) $${\mathrm{Threshold}}_{2}=W_{1}$$

**Figure 2:**
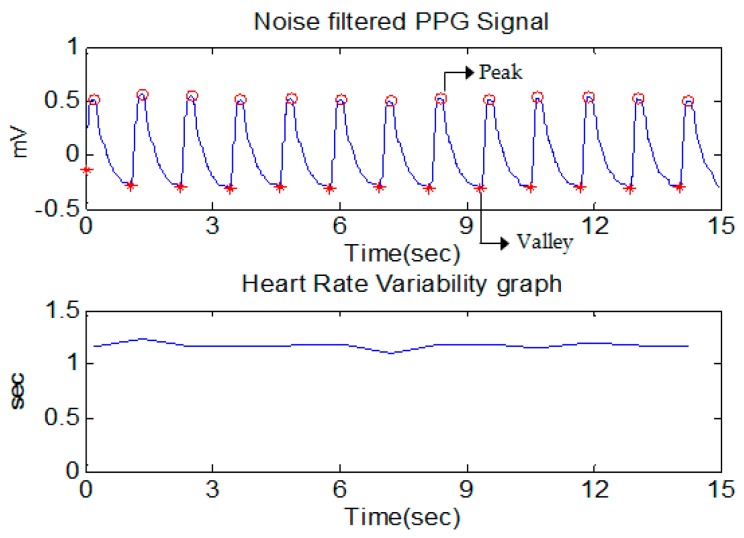
Peak and valley detection and Heart rate variability of PPG signal.

An interesting block containing the peak point was obtained by comparing the first threshold (${\mathrm{Threshold}}_{1})$ with the first moving average (${\mathrm{MA}}_{\mathrm{Peak}}$), and the erroneous block of interest generated by the noise was removed by comparing the size of each block of interest with the second threshold (${\mathrm{Threshold}}_{2}$). Finally, the peak data were detected by calculating the time index at which the maximum value in the block of interest is located, and the valley data were detected by calculating the time index where the minimum value between the two consecutive peaks is located. HRV can be measured by the variation in the peak-to-peak interval, which is the time interval between adjacent peaks. HRV is an important indicator for evaluating the sympathetic and parasympathetic activity of the autonomic nervous system [12]. Therefore, it is important to construct an accurate HRV measure to analyze the pain status by evaluating the autonomic nervous system activity. In fact, it is difficult to identify the correct peak-to-peak interval owing to various disturbances such as external noise, motion noise, and ectopic beats. To remove these artifacts, we used the filtering method proposed by Logier [13]. To illustrate the method, three threshold conditions were used for the peak-to-peak interval (Equations (7) and (8)). Peak-to-peak intervals belonging to one or more conditions are regarded as erroneous peak-to-peak intervals and are reconstructed at normal peak-to-peak intervals using linear interpolation. ${\mathrm{PPI}}_{i}$ is the peak-to-peak interval, and $m_{20}$ and $\sigma_{20}$ are the respective mean and standard deviation of the previous 20 peak-to-peak intervals. Peak, valley detection and heart rate variability of the PPG signal through the signal processing algorithm are shown in Figure 2.

(7) $$T_{1}={\mathrm{PPI}}_{i}<m_{20}-2\sigma_{20}{\;\mathrm{and}\;\mathrm{PPI}}_{i+1}>m_{20}+2\sigma_{20}$$

(8) $$T_{2}={\mathrm{PPI}}_{i}<0.75{\mathrm{PPI}}_{i-1}{\;\mathrm{or}\;\mathrm{PPI}}_{i+1}<0.75{\mathrm{PPI}}_{i-1}$$

(9) $$T_{3}={\mathrm{PPI}}_{i}>1.75{\mathrm{PPI}}_{i-1}$$

HRV results following filtering were utilized to extract the time domain and frequency domain of HRV. Because of different physical responses depending on pain status, we extracted the features according to the properties of the PPG that show the autonomic nervous system response. The extracted features are time-domain features from the geometry of the PPG signal, time domain features of HRV using statistical methods, and frequency domain features of HRV using spectrum analysis. This resulted in a total of 17 features. To extract the consecutive features from each signal data in the preoperative and immediate postoperative period, a 1-min sliding window method based on the PPG signal for 5 min, which is an appropriate time for short-term HRV analysis, was used [14].

The time-domain features from the geometry of the PPG signal were extracted from each 5-min window (Table 2). Instantaneous heart rate refers to the number of beats per minute calculated from the peak-to-peak interval. The instantaneous heart rate and the average heart rate can be estimated from Equations (10) and (11). N represents the number of samples of the instantaneous heart rate for 5 min.

(10) $$\mathrm{InstantaneousHR}=\frac{60\times \mathrm{Sampling}\;\mathrm{frequency}\;\left(300\;\mathrm{Hz}\right)}{\mathrm{Peaktopeak}\;\mathrm{Interval}}$$

(11) $$\mathrm{AverageHR}=\frac{1}{N}\overset{N}{\underset{i=1}{\sum }}{\left(\mathrm{InstantaneousHR}\right)}_{i}$$

Analysis of HRV in the time domain and frequency domain is used to assess not only heart disease but also stress status by estimating the periodic change of the heart rate [15]. The features of HRV are divided into the time domain and frequency domain. First, we extracted time domain features of HRV (Table 3).

In order to analyze HRV in the frequency domain, HRV time series data must be converted to the frequency domain. Therefore, the HRV power spectrum for specific frequency bands was calculated by converting it to the frequency domain using a fast Fourier transform (FFT) (Table 4).

The 17 features that were extracted in each 5-min window constitute the feature vector for generating the inputs of the classifier to identify the pain status. Biological signals such as PPG differ depending on the external environment and individual characteristics. The feature vectors extracted from the measured PPG signal include outliers. These outliers must be removed because they affect the performance of the pattern classifier. The median absolute deviation (MAD) method, which provides stronger performance for measures of dispersion, was used as an outlier removal method [16]. The MAD and the outlier removal method using it were modeled by Equations (12) and (13), respectively. A ± 2.5 MAD range at median value was set as the confidence interval and the outliers were removed.

(12) $${\mathrm{MAD}}_{j}=1.4826\times \mathrm{median}\left(\left|x_{i,j}-\mathrm{median}\left(x_{j}\right)\right|\right)$$

(13) $$\mathrm{Outlier}\to \{\begin{array}{c} x_{i,j}\leq \mathrm{median}\left(x_{j}\right)-2.5\times {\mathrm{MAD}}_{j}\\ x_{i,j}\geq \mathrm{median}\left(x_{j}\right)+2.5\times {\mathrm{MAD}}_{j} \end{array}$$

Moreover, the features extracted from the PPG signal have different ranges of values. Therefore, it is necessary to normalize all feature vectors to values between 0 and 1 to allow efficient learning of the pattern using the input vector of the classifier. The feature vectors with outliers removed for each feature were normalized through min-max normalization (Equation (14)).

(14) $$D_{i,j}^{\mathrm{Normalized}}=\frac{D_{i,j}-D_{i,j}^{\min}}{D_{i,j}^{\max}-D_{i,j}^{\min}}$$

Several classifiers have been used in pattern classification varying from linear discriminant analysis (LDA), artificial neural network (ANN), support vector machine (SVM), convolutional neural network (CNN), deep belief network (DBN), etc. In the present study, we used and evaluated 3 network architectures MLPNN, SVM, and DBN. MLPNN is a statistical learning algorithm that models biological brain structures. The MLPNN is composed of an input layer, at least one hidden layer, and an output layer according to a hierarchical structure [17]. The input layer and the output layer serve to receive the input data and output the result, and the hidden layer is used to calculate the data from the input layer as an active function and transmit it to the output layer. There are weights indicating the degree of connection between each layer. Weights are modified in the direction of decreasing the error between the output value according to the given input data and the target value desired by the user using a back-propagation algorithm with gradient descent [18]. SVM is a learning algorithm based on supervised learning, which is widely applied to binary classification problem and regression analysis [19]. Although the existing MLPNN and other neural networks are based on minimizing classification error, SVM attempts to maximize generalization ability to classify new data not used for learning [20]. DBN is a deep learning model that learns restricted boltzmann machine (RBM) by stacking several layers. DBN learning is divided into two stages. The first is the pre-training of unsupervised learning, which initializes all the weights and bias of the DBN. It plays a role of reconstruction, which is a process of estimating the probability distribution of input data. The second is fine-tuning of supervised learning, which uses the back propagation algorithm used in the MLPNN to fine-tune the weights and bias initialized in the pre-training to minimize the error between the target values of the given input data. It acts as a classifier. An ensemble model was applied to improve the performance of classifying pain status using these classifiers. The ensemble model is a model that combines multiple classifiers by a specific method to achieve better classification performance than a single classifier in classification problems [21]. In order for the ensemble model to perform well, it is important that each base classifier constructed has diversity. In other words, even if a few base classifiers are misclassified, if the rest of the base classifiers are correctly classified, the ensemble model is correctly classified through combining. We use the most typical bagging method among the ensemble models [22]. Bagging generates N different sets of training data by random sampling of the same size by bootstrap sampling, which is an extraction (duplication allowance) method from original training data. The base classifiers with diversity are generated by learning each base classifier using the generated different training data, and the results of classification for the original testing data are obtained. Finally, the final classification result is calculated by combining the classification result values of the base classifiers through a majority voting method among the combining methods. However, due to the random sampling through the bootstrap technique, some base classifiers improve final classification performance, but other base classifiers lower the final classification performance. Therefore, we proposed a selective ensemble model of the Hill-Climbing (HC) method. The selective ensemble model is not a combination of all the base classifiers generated in the model. Among these, the ensemble model is constructed by selecting only the base classifier which is expected to improve the performance in combining. At this time, a hill-climbing method was used to select only specific base classifiers [23].

## 3. Results

In this study, we compared the performance of the pain status classifiers developed through DBN, MLPNN, and SVM for the 2-class & 4-class pain classification according to the results of the NRS, and attempted to improve the performance by applying the bagging method to each classifier. The performance of pattern classifiers was evaluated with commonly used evaluation parameters such as accuracy, sensitivity, and specificity. These are statistical measures of the performance of a binary classification test. Sensitivity measures the proportion of positives that are correctly identified. Specificity measures the proportion of negatives that are correctly identified. Accuracy is expected to measure how well the test predicts both categories (positives and negatives). Therefore, we used accuracy to assess the performance of the pain status classifiers (Equation (15)). The process of calculating the accuracy of the classifiers uses the number of true positives (TP), true negatives (TN), false positives (FP), and false negatives (FN) in a confusion matrix.

(15) $$\mathrm{Accuracy}=\frac{\mathrm{TP}+\mathrm{TN}}{\mathrm{TP}+\mathrm{FP}+\mathrm{FN}+\mathrm{TN}}\times 100$$

### 3.1. Assessment of 2-Class Pain Status Classification Using Pattern Classification Algorithms

To compare the mean differences of the classification groups for the 17 extracted features according to pain status, we performed the Wilcoxon signed-rank test on the extracted features. The analysis revealed that there were statistically significant differences at the significance level p < 0.05 for 15 features excluding RMSSD and NN50 (Table 5). Features with insignificant differences for N and P (significance level p > 0.05) were excluded from the input variables to classify the 2-class pain status.

To evaluate the performance of the classifiers for N and P, the data were split into six groups; a 6-fold cross validation was used. The feature vectors of subjects were divided into a training set (1040 data points obtained from 52 subjects with 5-min windows each in two situations [preoperative period and immediate postoperative period]) and a validation set (260 data points obtained from 13 subjects with 5-min windows each in two situations [preoperative period and immediate postoperative period]), a testing set (260 data points obtained from 13 subjects with 5-min windows each in two situations [preoperative period and immediate postoperative period]). A training set was used for training, that is, to fit the parameters of a classifier. A validation set was used to tune the hyperparameters of a classifier. The testing set was never used in training. The testing set was used only to assess the performance of a classifier. The data used in the training, validation and testing process were divided evenly into two statuses to avoid focus on one status.

**Figure 3:**
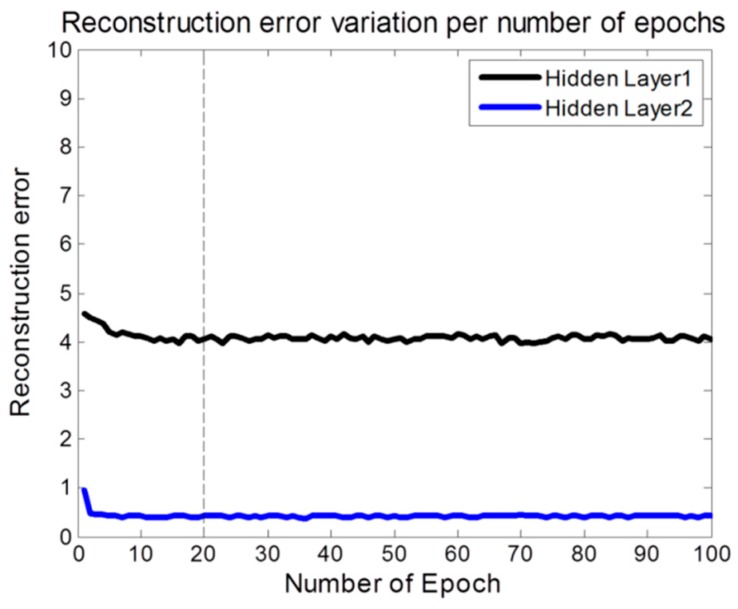
Reconstruction error variation per number of epochs.

We set the parameters of our DBN-based pain status classifier model (Table 6). The number of features represents the 15 features excluding RMSSD and NN50 through statistical significance evaluation above. Based on these, we tuned the number of epochs in the pre-training of DBN. As a result, the reconstruction error was gradually decreased and converged from 20 epochs in 2 hidden layers (Figure 3). We then tested the reconstruction error on the same conditions with the number of epochs in the fine-tuning of DBN. The performance of a single DBN model designed in this way was compared with a basic bagging model using DBN as a base classifier and a hill-climbing selective bagging model using DBN as a base classifier. The classifier of the ensemble model using the bagging differs according to the total number of base classifiers constituting the ensemble. The bootstrap sample size used for the bagging was the same as the original training data, and the total number of base classifiers was fixed at 50. The performance of the 2-class pain status classification for the three models using DBN was compared (Table 7).

We also constructed a model using MLPNN without RBM to compare the classification performance under the same conditions as DBN (Table 8). The performance of a single MLPNN model designed in this way was compared with a basic bagging model using MLPNN as a base classifier and a hill-climbing selective bagging model using MLPNN as a base classifier. The performance of the 2-class pain statuses classification for the three models using MLPNN was compared (Table 9).

To compare performance using another traditional model, SVM, we used a single SVM model, a basic bagging model using SVM as a base classifier, and a hill-climbing selective bagging model using SVM as a base classifier. In this case, we compared the performance by fixing the error penalty variable, C, to the default value of 1 and changing the parameter γ value of the RBF kernel considering the SVM model complexity (Table 10). According to the results, the optimal value of 0.05 was considered to be the γ value showing the highest classification accuracy of 82.12%. The performance of the 2-class pain statuses classification for the three models using SVM was compared (Table 11).

**Figure 4:**
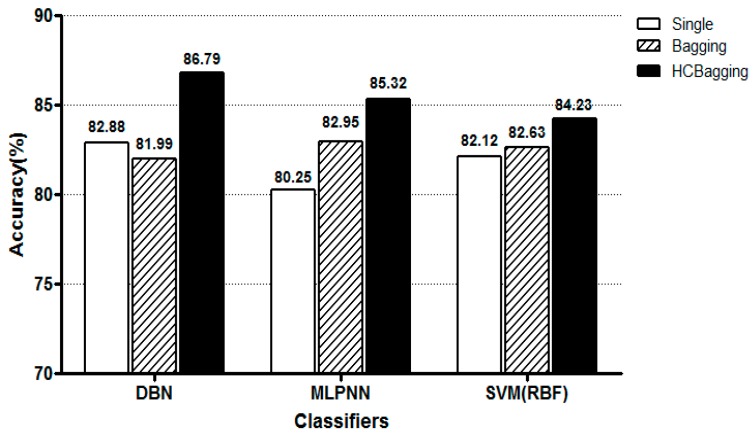
Evaluation of 2-class pain status classification performance of each pattern classification algorithm.

Figure 4 shows the classification accuracy of each model for the 2-class pain status classification. Here, Single refers to a single model, Bagging refers to a standard bating model, and HCBagging refers to the hill climbing selective bating model. It can be seen that the accuracy of each of the three classifiers is higher than that of each single model when applying the HC based selective bagging model. Also, DBN showed the best performance when comparing the accuracy of the three classifiers based on the selective bagging model.

**Figure 5:**
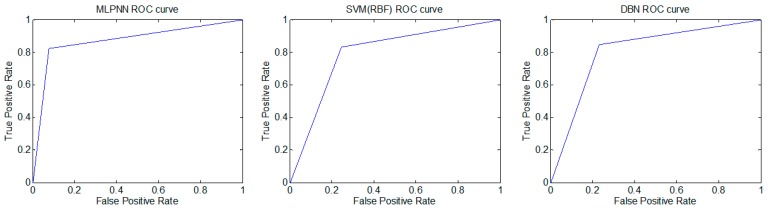
ROC curves for pain status classification using MLPNN, SVM(RBF) and DBN.

We performed Receiver operating characteristics (ROC) analysis on the MLPNN, SVM and DBN based pain status classifier of selective bagging model [24]. We observed the ROC curves for a randomly selected test group for 2-class pain status: Class 1 (no pain status) and Class 2 (pain status requiring active treatment) (Figure 5). The area under the ROC curve (AUC) was used to analyze the accuracy of the developed classifiers statistically. The classification performance was estimated using the AUC value. As the AUC value approaches 1, the classification model correctly classified the data. The results of ROC analysis for the MLPNN, SVM and DBN based pain status classifier of selective bagging model were shown in Table 12. The performance of the MLPNN based pain status classifier was revealed (AUC = 0.824 ± 0.029; mean ± s.d., n = 300, in a range of 0.820–0.827). The performance of the SVM based pain status classifier was revealed (AUC = 0.834 ± 0.029; mean ± s.d., n = 300, in a range of 0.831–0.837). Finally, The performance of the DBN based pain status classifier was revealed (AUC = 0.841 ± 0.039; mean ± s.d., n = 300, in a range of 0.836–0.845). The AUC values of the developed models were significantly higher than the theoretical baseline of 0.5 (one sample t-test, two-tailed, p < 0.0001, p < 0.0001, p < 0.0001, respectively for MLPNN, SVM, and DBN; n = 300). The developed models have good performance of pain status classification, and DBN of the three showed the highest AUC value.

### 3.2. Assessment of 4-Class Pain Status Classification Using Pattern Classification Algorithms

Depending on the results of the NRS we used to determine the actual patient’s pain status, we further classified the 4-class pain status—no pain status in the preoperative period and mild pain status, moderate pain status, severe pain status in immediate postoperative period. Therefore, we selected 93 subjects who reported mild, moderate, and severe pain in immediate postoperative period for analysis. Similar to the 2-class pain classification, we compared the performance of the pain status classifiers developed through DBN, MLPNN, and SVM for the 4-class pain classification. Table 13 shows the results of the significance tests for the 17 features extracted from the one-way ANOVA for the 4-class pain classification. The analysis showed that there were statistically significant differences at the significance level p < 0.05 for 17 features.

To evaluate the performance of the classifiers for no pain status, mild pain status, moderate pain status and severe pain status, the data were split into five groups; a 5-fold cross validation was used. The feature vectors of subjects were divided into a training set of 54 subjects and a validation set of 18 subjects, a testing set of 21 subjects. The data used in the training, validation and testing process were divided evenly into four statuses to avoid focus on one status.

The performance of a single DBN model designed to classify 4-class pain status was compared with a basic bagging model using DBN as a base classifier and a hill-climbing selective bagging model using DBN as a base classifier. The performance of the 4-class pain status classification for the three models using DBN was compared (Table 14).

The performance of a single MLPNN model designed to classify 4-class pain status was compared with a basic bagging model using MLPNN as a base classifier and a hill-climbing selective bagging model using MLPNN as a base classifier. The performance of the 4-class pain statuses classification for the three models using MLPNN was compared (Table 15).

The performance of a single SVM (RBF) model designed to classify 4-class pain status was compared with a basic bagging model using SVM (RBF) as a base classifier and a hill-climbing selective bagging model using SVM (RBF) as a base classifier. The performance of the 4-class pain statuses classification for the three models using SVM (RBF) was compared (Table 16).

**Figure 6:**
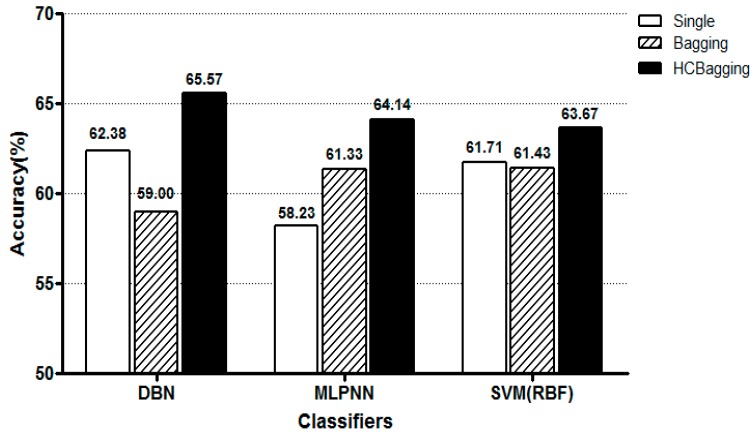
Evaluation of 4-class pain status classification performance of each pattern classification algorithm.

Figure 6 shows the classification accuracy of each model for the 4-class pain status classification. Likewise, the accuracy of each of the three classifiers was higher than that of each single model when the HC based selective bating model was applied. DBN showed the best performance based on the selective bating model.

## 4. Discussion

The present study was conducted on subjects who were conscious and as a preliminary study to develop a pain assessment system for patients in operation. The purpose of the present study was to design a pain classifier with high accuracy in classifying the status of conscious patients’ pain. The actual status of conscious subjects’ pain was determined using the numeric rating scale (NRS), and the status of pain was classified on the basis of the determined pain status. Accurate classification of pain status is difficult because humans have different sensitivities to painful stimuli according to an individual’s physical constitution and inclination. In the present study, photoplethysmography signals depending on the preoperative and postoperative pain status of conscious patients were obtained for the objective determination of pain status, and the characteristics of the signals were extracted by accurate and continuous signal analysis based on a sliding window method. The extracted characteristics were learned by multilayer perceptron neural network (MLPNN) and support vector machine (SVM), which are machine learning methods, and deep belief network (DBN), which is a deep learning method to determine the pain status. The classification performance was improved by applying a selective bagging model.

The results showed that the classification performance was better with the DBN method than with the MLPNN method, by which local solutions were obtained, or with the SVM, which is often employed to solve classification problems, by about 2.6% and 0.7%, respectively. It is obvious from ROC analysis also that the all three models have good performance of pain status classification, but DBN showed the highest AUC value.

Application of the standard bagging model decreased the classification accuracy in the DBN method in comparison to the application of a single model classifier. This finding may be the result of the random sampling based on the bootstrap technique. Therefore, when a selective bagging model was applied by constituting an ensemble model consisting of the selected base classifiers that were expected to improve the performance, the classification performance improved in comparison with the individual single model-based classifiers. The highest accuracy (86.79%) was observed when the DBN was used. We could confirm that the 4-class pain status classification is less accurate than the 2-class pain status classification. This is due to the fact that there is a difference in the number of training data points in the classification class because the data points by the NRS for the pain status are uneven.

In the present study, a probabilistic pain status classifier was designed to learn the complicated nonlinear correlations between the characteristics extracted using photoplethysmography and the NRS-based pain status. As the DBN is based on the restricted Boltzmann machine (RBM), the problem of local optimization found in the MLPNN may be reduced. However, the parameters used were empirically set up in the process of designing the DBN. Further optimization of the parameters may improve the pain status classification performance. In addition, better results may be obtained through the learning of the classifiers with more subjects in each of the NRS levels. Finally, owing to the large differences in the biological signals among individuals, considering not just one type of signal but the interactions among two or more signals may allow for more accurate evaluation of patients’ pain status. The results of the present study may assist in development of a pain status evaluation system based on photoplethysmography signals depending on the pain status of patients undergoing surgery.

## 5. Conclusions

This study was conducted as a preliminary study to develop a pain assessment system for patients undergoing surgery. It is difficult to judge the pain status because the sensitivities to the stimuli are different according to the constitution and tendency of the individual, and the judgment using the existing clinical signs is a subjective factor. In order to determine the objective pain status, we designed a pain status classifier applying DBN based on photoplethysmography signal affected by the autonomic nervous system, and improved classification performance by applying a bagging ensemble technique. As a result of comparison with MLPNN and the SVM method to evaluate the DBN-based pain classifier applying the selective bagging model, the highest classification accuracy was obtained when DBN was used in all of the 2-class and 4-class pain classifications. Compared with the DBN-based pain status classifier of a single model, the performance was higher when selective bagging was applied. The results of this study will contribute to the development of a pain assessment system based on photoplethysmography signal according to the pain status of the operation patients in the future. In future studies, it is necessary to study the pain status based on the data obtained by the surgical stimulation for the actual anesthetized patient in order to develop an evaluation system of the pain condition in the actual operating environment. In addition, The further studies can contribute to the determination of health condition if we study the pain that occurs in daily life applied to the general person rather than the patient.

## Figures and Tables

**Table 1:** Numerical rating scales of pain status in the immediate postoperative period.

NRS (Pain Level)	No Active Treatment Required				Active Treatment Required						
	None	Mild			Moderate			Severe			
	0	1	2	3	4	5	6	7	8	9	10
Subjects in the immediate postoperative period (N = 100)	7	2	4	9	10	20	7	17	17	5	2

**Table 2:** Time-domain features from geometry of PPG signal.

Features	Description
Pulse height	Average of the distances between Peaks and Valleys in a 5-min window
Rise time	Average of the time it takes to rise from Valley to Peak in a 5-min window
Fall time	Average of the time it takes to fall from Peak to Valley in a 5-min window
Average heart rate	Average of the instantaneous heart rate in a 5-min window
$T_{3}={\mathrm{PPI}}_{i}>1.75{\mathrm{PPI}}_{i-1}$	(10)

**Table 3:** Time domain features of HRV.

Features	Description
AVNN	Average of the Peak to Peak (NN) intervals observed in a 5-min window
SDNN	Standard deviation of the Peak to Peak intervals observed in a 5-min window
RMSSD	Root mean square difference of successive Peak to Peak intervals in a 5-min window
NN20	Number of pairs of successive Peak to Peak intervals that differ by more than 20 ms in a 5-min window
pNN20	The proportion of NN20 divided by the total number of Peak to Peak intervals in a 5-min window
NN50	Number of pairs of successive Peak to Peak intervals that differ by more than 50 ms in a 5-min window
pNN50	The proportion of NN50 divided by the total number of Peak to Peak intervals in a 5-min window

**Table 4:** Frequency domain features of HRV.

Features	Description
VLF power	Power in very low frequency range (0.003–0.04 Hz)
LF power	Power in low frequency range (0.04–0.15 Hz)
HF power	Power in high frequency range (0.15–0.4 Hz)
Total power	Power in very low frequency range (0.003–0.4 Hz)
LF/HF Ratio	LF/HF
Respiratory rate power	Maximum power in frequency range (0.1–0.25 Hz)

**Table 5:** Statistical significance of the extracted features.

Feature	Preoperative Period [Mean ± SD]	Immediately Postoperative Period [Mean ± SD]	p-Value	Significant
Pulse Height	0.908 ± 0.206	0.673 ± 0.489	<0.0001	Yes
Rise time	0.219 ± 0.05	0.227 ± 0.046	<0.0001	Yes
Fall time	0.700 ± 0.128	0.660 ± 0.115	<0.0001	Yes
Average heart rate	66.32 ± 10.30	69.66 ± 10.28	<0.0001	Yes
AVNN	0.928 ± 0.145	0.883 ± 0.130	<0.0001	Yes
SDNN	0.041 ± 0.016	0.039 ± 0.019	<0.0001	Yes
rMSSD	0.031 ± 0.016	0.032 ± 0.019	0.6798	No
NN20	133.1 ± 61.65	104.3 ± 65.65	<0.0001	Yes
pNN20	42.61 ± 22.02	31.39 ± 20.28	<0.0001	Yes
NN50	32.12 ± 31.33	30.05 ± 29.21	0.1573	No
pNN50	10.02 ± 9.808	9.069 ± 8.903	0.0279	Yes
VLF power	0.517 ± 0.374	0.453 ± 0.399	<0.0001	Yes
LF power	0.298 ± 0.220	0.209 ± 0.190	<0.0001	Yes
HF power	0.256 ± 0.204	0.214 ± 0.190	<0.0001	Yes
Total power	1.145 ± 0.770	1.053 ± 0.894	0.0005	Yes
LF/HF ratio	1.647 ± 1.202	1.190 ± 0.871	<0.0001	Yes
Respiratory rate power	7.958 ± 6.532	6.060 ± 5.620	<0.0001	Yes

**Table 6:** Parameters of DBN-based pain status classifier model.

Structure from Input Layer to Output Layer	15-6-6-2
Number of features	15
Number of hidden layers	2
Number of hidden neurons on the hidden layers	6
Learning rate for weight	0.08
Learning rate for biases of visible units	0.08
Learning rate for biases of hidden units	0.08
Number of batch size	104
Momentum rate	0.9
Number of epoch in the pre-training	10 to 100
Number of epoch in the fine-tuning	100 to 800
Weight decay	0.00029
Activation function	sigmoid function

**Table 7:** Performances of the 2-class pain status classification for the three models using DBN.

	Input Vector	15 Features
Classifier Model		
Single model		82.88
basic bagging model		81.99
selective bagging model		86.79

**Table 8:** Parameters of MLPNN-based pain status classifier model.

Structure from Input Layer to Output Layer	15-6-6-2
Number of features	15
Number of hidden layers	2
Number of hidden neurons on the hidden layers	6
Learning rate for hidden layers	0.08
Number of batch size	104
Number of epoch in the training	800
Weight decay	0.00029
Activation function	sigmoid function

**Table 9:** Performances of the 2-class pain status classification for the three models using MLPNN.

	Input Vector	15 Features
Classifier Model		
Single model		80.25
basic bagging model		82.95
selective bagging model		85.32

**Table 10:** Performances of the 2-class pain status classification by γ value using SVM (RBF).

	Input Vector	15 Features
γ		
0.001		70.51
0.005		77.50
0.01		79.81
0.05		82.12
0.1		80.71
0.5		75.32
1		72.69

**Table 11:** Performances of the 2-class pain status classification for the three models using SVM.

	Input Vector	15 Features
Classifier Model		
Single model		82.12
basic bagging model		82.63
selective bagging model		84.23

**Table 12:** ROC analysis of the pain status on the developed models.

Metrics	MLPNN	SVM(RBF)	DBN
Mean AUC	0.824	0.834	0.841
Standard deviation AUC	0.029	0.029	0.039
Lower limit of 95% Confidence interval	0.820	0.831	0.836
Upper limit of 95% Confidence interval	0.827	0.837	0.845

**Table 13:** Statistical significance of the extracted features for 4-class pain status classification.

Feature	p-Value	F-Value	Significant
Degrees of Freedom (dF) (Between Groups = 3, Within Groups = 1856)			
Pulse Height	<0.0001	72.39	Yes
Rise time	<0.0001	10.11	Yes
Fall time	<0.0001	28.66	Yes
Average heart rate	<0.0001	21.50	Yes
AVNN	<0.0001	23.37	Yes
SDNN	<0.0001	17.07	Yes
rMSSD	<0.0001	10.43	No
NN20	<0.0001	45.38	Yes
pNN20	<0.0001	58.53	Yes
NN50	<0.0001	22.20	Yes
pNN50	<0.0001	22.63	Yes
VLF power	<0.0001	10.61	Yes
LF power	<0.0001	61.75	Yes
HF power	<0.0001	26.40	Yes
Total power	<0.0001	25.77	Yes
LF/HF ratio	<0.0001	32.53	Yes
Respiratory rate power	<0.0001	37.13	Yes

**Table 14:** Performances of the 4-class pain status classification for the three models using DBN.

	Input Vector	17 Features
Classifier Model		
Single model		62.38
basic bagging model		59.00
selective bagging model		65.57

**Table 15:** Performances of the 4-class pain status classification for the three models using MLPNN.

	Input Vector	17 Features
Classifier Model		
Single model		58.23
basic bagging model		61.33
selective bagging model		64.14

**Table 16:** Performances of the 4-class pain status classification for the three models using SVM.

	Input Vector	17 Features
Classifier Model		
Single model		61.71
basic bagging model		61.43
selective bagging model		63.67

## References

[1] Raj P.P. (2007). Taxonomy and classification of pain. The Handbook of Chronic Pain.

[2] Chapman C.R., Tuckett R.P., Song C.W. (2008). Pain and stress in a systems perspective: Reciprocal neural, endocrine, and immune interactions. J. Pain.

[3] Desborough J. (2000). The stress response to trauma and surgery. Br. J. Anaesth..

[4] Holte K., Kehlet H. (2002). Epidural anaesthesia and analgesia–effects on surgical stress responses and implications for postoperative nutrition. Clin. Nutr..

[5] Gruenewald M., Ilies C. (2013). Monitoring the nociception–anti-nociception balance. Best Pract. Res. Clin. Anaesthesiol..

[6] Hartrick C.T., Kovan J.P., Shapiro S. (2003). The numeric rating scale for clinical pain measurement: A ratio measure?. Pain Pract..

[7] Kaul H., Bharti N. (2002). Monitoring depth of anaesthesia. Indian J. Anaesth..

[8] Asada H.H., Shaltis P., Reisner A., Rhee S., Hutchinson R.C. (2003). Mobile monitoring with wearable photoplethysmographic biosensors. IEEE Eng. Med. Biol. Mag..

[9] Ferreira-Valente M.A., Pais-Ribeiro J.L., Jensen M.P. (2011). Validity of four pain intensity rating scales. Pain.

[10] Allen J. (2007). Photoplethysmography and its application in clinical physiological measurement. Physiol. Meas..

[11] Elgendi M., Norton I., Brearley M., Abbott D., Schuurmans D. (2013). Systolic peak detection in acceleration photoplethysmograms measured from emergency responders in tropical conditions. PLoS ONE.

[12] Cowan M.J. (1995). Measurement of heart rate variability. West. J. Nurs. Res..

[13] Logier R., De Jonckheere J., Dassonneville A. (2004). An efficient algorithm for RR intervals series filtering. Conf. Proc. IEEE Eng. Med. Biol. Soc..

[14] Variability H.R. (1996). Standards of measurement, physiological interpretation, and clinical use. Task Force of the European Society of Cardiology and the North American Society of Pacing and Electrophysiology. Circulation.

[15] Kim K.-S., Shin S.-W., Lee J.-W., Choi H.-J. (2008). The assessment of dynamic mental stress with wearable heart activity monitoring system. Trans. Korean Inst. Electr. Eng..

[16] Leys C., Ley C., Klein O., Bernard P., Licata L. (2013). Detecting outliers: Do not use standard deviation around the mean, use absolute deviation around the median. J. Exp. Soc. Psychol..

[17] Hornik K., Stinchcombe M., White H. (1989). Multilayer feedforward networks are universal approximators. Neural Netw..

[18] Trafalis T.B. (1995). Neural Networks: Algorithms, Applications and Programming Techniques.

[19] Cortes C., Vapnik V. (1995). Support-vector networks. Mach. Learn..

[20] Burges C.J.C. (1998). A tutorial on support vector machines for pattern recognition. Data Min. Knowl. Discov..

[21] Dietterich T.G. (1997). Machine-learning research. AI Mag..

[22] Breiman L. (1996). Bagging predictors. Mach. Learn..

[23] Li K., Liu Z., Han Y. (2012). Study of selective ensemble learning methods based on support vector machine. Phys. Procedia.

[24] Fawcett T. (2006). An introduction to ROC analysis. Pattern Recognit. Lett..

